# Association of peripheral artery disease and chronic limb-threatening ischemia with socioeconomic deprivation in people with diabetes: A population data-linkage and geospatial analysis

**DOI:** 10.1177/1358863X20981132

**Published:** 2021-01-25

**Authors:** Joanne E Hurst, Peta Ellen Tehan, Keith Hussey, James Woodburn

**Affiliations:** 1 School of Health and Life Sciences, Glasgow Caledonian University, Glasgow, Scotland, UK; 2 School of Health Sciences, Faculty of Health & Medicine, University of Newcastle, Ourimbah, NSW, Australia; 3 Department of Vascular Surgery, Queen Elizabeth University Hospital, Glasgow, Scotland, UK

**Keywords:** chronic limb-threatening ischemia (CLTI), diabetes, geospatial mapping, health disparities, peripheral artery disease (PAD), spatial clustering

## Abstract

The association between the prevalence and geographical distribution of peripheral artery disease (PAD) and chronic limb-threatening ischemia (CLTI) in patients with diabetes in the context of socioeconomic deprivation is not well understood. We undertook a retrospective cohort study of 76,307 people with diabetes admitted as a hospital inpatient in a large Scottish health administrative area. Utilising linked health records, we identified diagnoses of PAD and/or CLTI and their distribution using small area cartography techniques according to multiple deprivation maps. Spatial autocorrelation techniques were applied to examine PAD and CLTI patterning. Association between crude inpatient prevalence-adjusted outcome rates and exposure to social deprivation were determined. We found crude prevalence-adjusted rates of 8.05% for PAD and 1.10% for CLTI with a five- to sevenfold difference from the least to most deprived regions. Statistically significant hot spots were found for PAD (*p* < 0.001) and CLTI (*p* < 0.001) in the most deprived areas, and cold spots for PAD (*p* < 0.001) but not CLTI (*p* = 0.72) in the least deprived areas. Major health disparities in PAD/CLTI diagnoses in people with diabetes is driven by socioeconomic deprivation.

## Introduction

Peripheral artery disease (PAD) and chronic limb-threatening ischemia (CLTI), can lead to impaired quality of life, increased mortality, delayed wound healing and increased risk of lower extremity amputation (LEA).^1–4^ People with diabetes are particularly vulnerable with higher prevalence of PAD and CLTI, which can be challenging to diagnose, and these patients generally require more complex interventions and management.^5–9^ Whilst risk factors for PAD and CLTI, such as diabetes, end-stage renal disease, and coronary artery disease are well established, the relationship between PAD, CLTI, and multiple deprivation is more controversial.^10–12^

Multiple deprivation is inextricably linked to general health outcomes,^13^ with higher incidences of most diseases occurring in more deprived populations.^14^ It is proposed that these populations have more barriers in accessing healthcare, are less likely to engage in healthy behaviours such as physical activity, subsequently have higher rates of obesity, and are more likely to undertake damaging health behaviours such as smoking.^13^ Large geographical health administration areas in NHS England demonstrate prevalence variation for LEA and revascularisation but no association with socioeconomic deprivation.^11^ Conversely, increased deprivation associated with an increased likelihood of LEA secondary to PAD can be demonstrated when studied at a local level.^15^ This has previously been established for people with diabetes, and outcomes of diabetic foot ulceration, LEA, and subsequent mortality are associated with multiple deprivation, with a four- to fivefold variation in rates of LEA from the most to least deprived neighbourhoods.^16^ The geographical patterning of PAD and CLTI in people with diabetes and its association with multiple deprivation has not been fully investigated. In order to inform future targeted interventions and health resource allocation, it is important to understand neighbourhood clustering. We hypothesised that individuals with diabetes and an inpatient discharge diagnosis of PAD and/or CLTI would be associated at nearer localities with similar levels of social disadvantage than PAD and/or CLTI events at areas further apart. Therefore, the aim of this study was to investigate the spatial clustering of PAD and/or CLTI in patients with diabetes who have been admitted as a hospital inpatient and, secondly, to determine the association of PAD and/or CLTI with multiple deprivation.

## Methods and materials

### Data sources

This retrospective cohort study was performed using data linkage and geospatial mapping carried out within the health administrative boundaries of NHS Greater Glasgow and Clyde Health Board. The central linkage point was through The Scottish Care Information – Diabetes Collaboration (SCI-Diabetes), a live clinical registry for the people in Scotland with a diagnosis of diabetes. Datasets were linked at the individual patient level using the Community Health Index (CHI) number – a distinct patient identifier used in Scottish healthcare records. We used SCI-Diabetes and the national inpatient hospital admissions dataset Scottish Morbidity Record (SMR-01) to gather demographic and clinical data from a fully assimilated electronic patient record. We also used the National Records of Scotland (NRS) to obtain geocoding information. The data were pseudo-anonymised before databases were accessed through a protected virtual analysis environment. The data linkage was provided by NHS Greater Glasgow Clyde Safe Haven.

### Ethical approval

We obtained peer review, Safe Haven review, Local Privacy Advisory Committee and Caldicott Guardian approvals (reference: GSH/16/DI/002) for the study.

### Cohort population

Data extraction was undertaken in November of 2016, the study end point was established at the absolute final stage of updated data entry and full coverage of SMR-01 was recognised. This was documented in August 2016. We included all inpatients who matched in the SCI-Diabetes database registered within NHS Greater Glasgow and Clyde from 1 January 2002. There was a total of 877,124 SMR-01 admissions recorded over the study period. A diagnosis of diabetes was ensured by cross-matching those with a recorded diabetes type (based on webforms from SCI-Diabetes, in conjunction with diagnostic evidence drawn from practice systems) with those patients who had a PAD and/or CLTI SMR-01 diagnosis. Any individual admitted under the age of 18 was excluded in the spatial model as they were less likely to experience the diagnoses investigated.

### Study geographical area

The study was conducted within the health administration area of NHS Greater Glasgow and Clyde. This geographical area comprises six local authorities with a population of 1,169,110 (21.6% of the Scottish population). Complete and reliable geographical coverage of an inpatient discharge diagnosis of PAD and/or CLTI was undertaken over a 13.6-year period by linking SMR-01 diagnoses with residential location, based on NRS data.

### Study variables

From SMR-01 and SCI-Diabetes, we described the study population by ascertaining age (at point of inpatient admission), sex, diabetes type, and ethnicity. Individuals with a diagnosis of PAD and/or CLTI events were extracted from SMR-01 using the 10th revision of the *International Statistical Classification of Diseases and Related Health Problems* (ICD-10; WHO, Geneva, Switzerland) codes from up to a possible six discharge diagnoses per admission. The following ICD-10 codes were selected for analysis for PAD: I70.2; I70.20; I70.21; I73.9; I73.8; E11.5; E10.5; I70.90; I179.3; and/or CLTI: I74.0; I74.1; I74.3; I73.4; I74.5; I74.8; I74.9. The diagnostic coding pertains to a clinical examination augmented by either noninvasive vascular assessment or cross-sectional arterial imaging. We used the Scottish Index of Multiple Deprivation (SIMD) 2016 score, extracted at point of inpatient admission, to explore each patient’s level of exposure to social deprivation (https://www2.gov.scot/Topics/Statistics/SIMD). SIMD is a tool which links similar regions of multiple deprivation. Each inpatient with diabetes was allocated a data zone during data linkage. It is calculated using the patient’s most recent census and postcode information from the NRS record linked with the SMR-01. The capture of SIMD comprises seven main domains including: health; income; employment rates; crime rates; standard of housing; education attainment; and access to services – which combines a total of 38 individual measurements to calculate the small area geographies deprivation score. The health administrative region of NHS Greater Glasgow and Clyde encompassed 1460 individual data. Deprivation scores were ranked for all national small area geographies. Each individual was given a quintile score: 20% of the most deprived areas were represented by quintile 1 zones and the 20% least deprived areas were apportioned to quintile 5 zones (see online supplementary material Figure 1). The total number of patients with diabetes admitted to hospital with an SMR-01 event formed the denominator. There were two numerators required to undertake the analysis: (a) those individuals with diabetes and a discharge diagnosis of PAD; and/or (b) those with diabetes and a discharge diagnosis of CLTI. Each inpatient with diabetes was geocoded by their allocated data zone number during data linkage to the NRS dataset and mapped to the SIMD 2016 shape file.

### Statistical analysis

We described the study population and its demographic and clinical characteristics using mean, SD, discrete values, and percentages from SMR-01 and SCI-Diabetes. Output hot spot maps were created to demonstrate the spatial clustering of PAD and/or CLTI distribution across the included data zones. The patient’s data zone number was extracted and linked to health records matching the shape file for the 2016 SIMD map. The population with diabetes who had an inpatient episode of care for each data zone were identified, revealing the crude inpatient prevalence-adjusted rates for PAD and/or CLTI rates which were calculated over the study period.

The spatial autocorrelation statistics were undertaken at the level of the small area geographies, each data zone comprising a mean of 760 individuals. We conducted spatial autocorrelation using a hot spot cluster analysis Getis–Ord Gi*.^17^ In the spatial statistical model, it was essential to conceptualise two of the spatial parameters:

1. In the model, there are differing sizes of polygons for each data zone. It is evident that the data zones were larger towards the administrative margins, representing less densely populated data zones, and smaller heading centrally, towards the more urban regions in Glasgow city. Accordingly, we adjusted for this using a theoretical fixed distance band method, which considered the differing polygon dimensions.
2. We modified the model for data zones sharing the same boundaries. This was achieved in the spatial model by including contiguity edges and corners, so that polygons with a shared boundary or corner were entered in isolation for each calculation of the cluster analysis.

This clustering analysis utilises the G_i_* statistic which produces a *z*-score and which assumes a normal distribution across all the data zones. It specifically examines each data zone to identify where high and low values cluster spatially in relation to their neighbouring polygons.^17^ Any patterning identified with the formation of clusters with 90%, 95%, 99% significance levels, from a two-tailed dispersion, stipulate statistically significant clustering of polygons. Within the SIMD maps, the output G_i_* *z*-score was visualised through colour coding (red indicating hot spots; blue indicating cold spots; yellow, not significant). The attribute mapping outputs were examined to investigate the dispersal of hot and cold spot patterning across SIMD quintiles using a one-sample χ^2^ test. A *p*-value less than 0.05 was used as a cut-off for statistical significance. Geospatial mapping analyses were undertaken using ArcGIS 10.4 Geostatistical Analyst (ESRI, Redlands, CA, USA). Data cleaning and all other analyses were conducted using IBM SPSS Statistics Version 27 (IBM Corp., Armonk, NY, USA).

## Results

We extracted and linked the health records of 76,307 patients with diabetes admitted to secondary care. The mean age of the study population was 66.3 years (SD 15.3 years), 53% were male, 72.3% were of white Scottish/British ethnicity, 90.3% had type 2 diabetes, and 41.5% were non-smokers with a mean body mass index of 33.5 kg/m^2^ (Table 1).

**Table 1.** Demographic and clinical characteristics of the total population with diabetes and PAD and/or CLTI.

Demographic characteristics	Inpatients with diabetes admitted to secondary care (denominator) *n* = 76,307	PAD diagnosis (numerator a) *n* = 6144	CLTI diagnosis (numerator b) *n* = 841
Mean age, years	66.3 (SD 15.3)	68.6 (SD 11.8)	67.5 (SD 11.4)
Sex			
Male	40,466 (53.0)	3250 (52.9)	515 (61.2)
Female	35,841 (47.0)	2894 (47.1)	326 (38.8)
Ethnicity			
White Scottish/British	54,806 (71.8)	4316 (70.2)	600 (71.3)
Any other White ethnic group or White non-British	6380 (8.4)	783 (12.7)	119 (14.1)
Asian, Asian Scottish, Asian British	4502 (5.9)	154 (2.5)	17 (2.0)
African, Caribbean, or Black	472 (0.6)	5 (< 0.0)	–
Any other ethnic group	1318 (1.7)	65 (1.1)	11 (1.3)
Not known/refused	3114 (4.1)	269 (4.4)	35 (4.2)
Missing	5715 (7.5)	522 (9.0)	59 (7.0)
Disease type			
Type 1	6307 (8.3)	684 (11.1)	84 (10.5)
Type 2	68,907 (90.3)	5390 (87.7)	747 (88.8)
Other	1093 (1.4)	70 (1.2)	10 (1.2)
Smoking status			
Never smoked	31,716 (41.5)	1442 (23.5)	307 (36.5)
Current smoker	14,155 (18.6)	1194 (19.4)	203 (24.1)
Ex-smoker	19,820 (26.0)	1572 (25.6)	271 (32.2)
Not known/declined	490 (0.6)	22 (0.4)	6 (0.7)
Missing	10,126 (13.3)	1914 (31.2)	54 (6.4)
Mean BMI (kg/m^2^)	33.5 (SD 7.4)	32.5 (SD 7.1)	32.4 (SD 6.9)
Mean BMI	15,069 (19.7)	1994 (32.5)	70 (8.3)
SIMD quintile			
Q1 (most deprived)	31,526 (41.3)	3050 (49.6)	477 (56.7)
Q2	14,465 (19.0)	1212 (19.7)	145 (17.2)
Q3	10,257 (13.4)	757 (12.5)	98 (11.7)
Q4	8553 (11.2)	582 (9.5)	60 (7.1)
Q5 (least deprived)	10,444 (13.7)	541 (8.8)	60 (7.1)
Missing	1062 (1.4)	2 (< 0.0)	1 (0.1)

Values presented as frequencies (%) unless stated otherwise.

BMI, body mass index; CLTI, chronic limb-threatening ischaemia; PAD, peripheral artery disease; Q, deprivation quintile; SIMD, Scottish Index of Multiple Deprivation.

**Figure 1.**
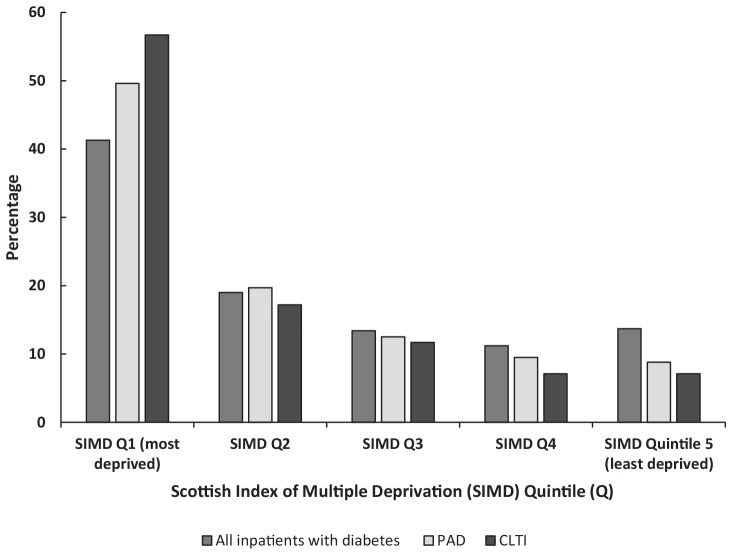
Distribution of inpatients with diabetes and diagnoses of PAD and/or CLTI across SIMD quintiles. CLTI, chronic limb-threatening ischaemia; PAD, peripheral artery disease.

Exposure to multiple deprivation was common, with 41.3% of individuals distributed in SIMD Q1 (most deprived) and 13.7% in SIMD Q5 (least deprived) (Figure 1). Over the 13.6-year study period, PAD was identified in 6144 (8.05%) individuals and CLTI in 841 (1.10%) individuals.

**Figure 2.**
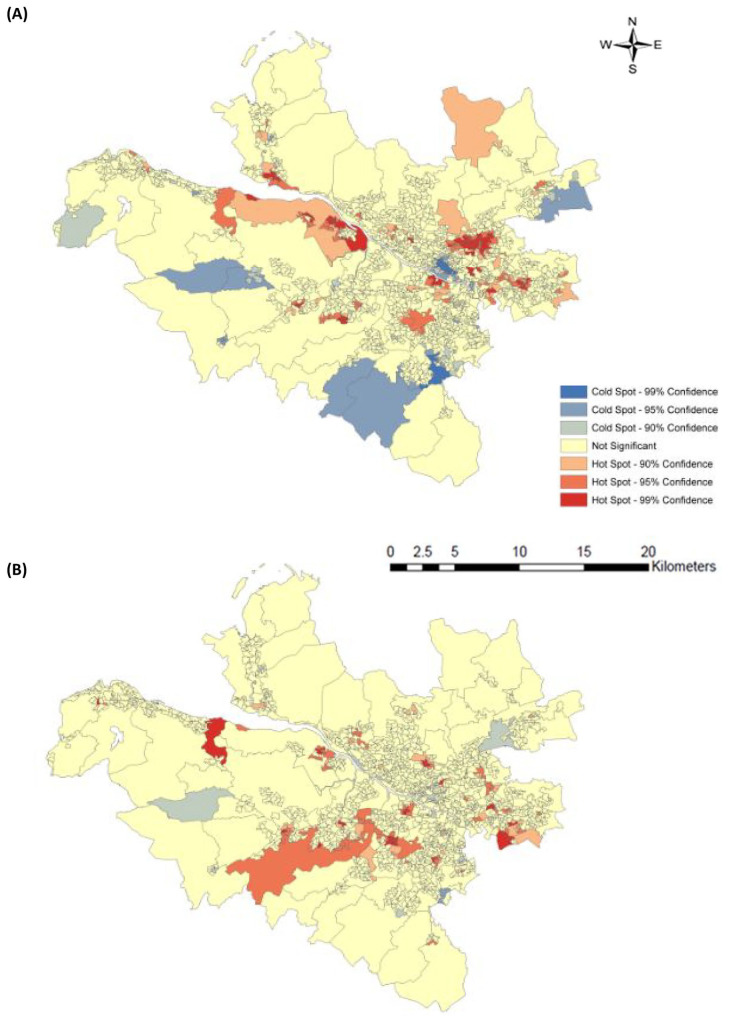
Map of spatial distribution of patients with diabetes and discharge diagnosis of (A) PAD and/or (B) CLTI using hot spot analysis. *Data source*: The 2016 Scottish Index of Multiple Deprivation (SIMD) map shape file is available on the Scottish Government website: https://www2.gov.scot/Topics/Statistics/SIMD. CLTI, chronic limb-threatening ischaemia; PAD, peripheral artery disease. Note – This figure is in colour online.

A statistically significant trend of a higher distribution of PAD diagnosis (χ^2^ [*df*] value, *p* value), χ^2^[4] 3607.2, *p* < 0.001) and/or CLTI diagnosis (χ^2^[4] 739.5, *p* < 0.001) in SIMD Q1 was found. We identified statistically significant geospatial patterns from the spatial autocorrelation analysis, resulting in clustering of hot spots (high-high prevalence) and cold spots (low-low prevalence) for PAD and/or CLTI from 2002 to 2016). Of total data zones, we found 162/1460 (11.1%) formed hot spot and 84 (5.8%) formed cold spot data zones for PAD diagnosis; 126 (8.6%) formed hot spot and 26 (1.6%) formed cold spot data zones for CLTI diagnosis. Figure 2 provides a chromatic representation of the spatial patterning of hot and cold spot clusters across the health administrative region.

**Figure 3.**
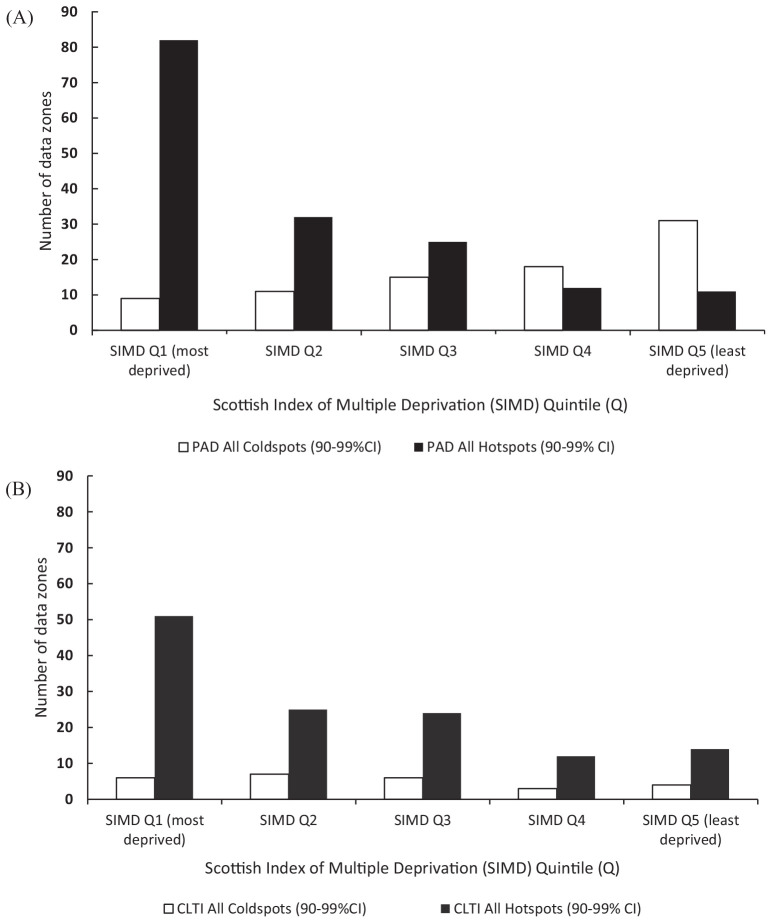
Distribution of hot and cold spot clusters across SIMD quintiles for (a) peripheral artery disease (PAD) and (b) chronic limb-threatening ischemia (CLTI).

Neighbouring data zones show similarly high or low prevalence for both diagnoses. We identified neighbouring clustering of high prevalence (hot spot) data zones. Hot spot clusters formed in the South and East regions of the more urban localities, with some overlapping data zones into the Inverclyde region across the health board for both PAD and CLTI. There were fewer formations of cold spots, particularly for CLTI. Despite this, there are some corresponding areas highlighted by their appearance in the west end of Glasgow – in the localities directly north of the River Clyde from the city centre. The distribution of hot spots was higher within SIMD quintile 1 (most deprived) for PAD (χ^2^4 104.6, *p* < 0.001) and CLTI (χ^2^4 38.4, *p* < 0.001). Although there were more cold spots forming in SIMD quintile 5 (least deprived) for PAD (χ^2^4 17.91, *p* = 0.001), the same trend was not identified for CLTI (χ^2^4 2.08, *p* = 0.72), as shown in Figure 3.

For both PAD and CLTI diagnoses, the majority of hot spot clusters (70.4% and 60.3%, respectively) were found across quintiles 1 and 2. In addition, a lower dispersal of hot spots for both event outcomes were detected in the less deprived SIMD quintiles (3–5). Despite this, the association with cold spots clustering in less deprived data zones were less pronounced. For PAD, the majority congregated in quintile 5 (36.9%) and were dispersed fairly equally across quintiles 2–4, with a lower distribution in quintile 1 (10.7%). However, this was not apparent for CLTI, as no real trend could be observed.

## Discussion

In this study, we uncovered a 5.6–7.9-fold difference in the crude prevalence-adjusted rates of people with diabetes admitted to secondary care with a discharge diagnosis of PAD and/or CLTI between the least and most deprived regions. Further, areas of relative high prevalence cluster in areas of high social deprivation associated with post-industrial decline and, conversely, for PAD, areas of low prevalence in areas of least deprivation.

The association between socioeconomic status and PAD and CLTI is not well established.^18^ We conducted this study in the west of Scotland, specifically Greater Glasgow and Clyde, which hosts some of the most deprived small area localities in Scotland.^19^ We have previously demonstrated an association between social deprivation and diabetic foot ulceration and LEA in a similar cohort within the same geographical region.^16^ Since PAD is a recognised risk factor for these diabetic foot complications, we were unsurprised to find similar inequalities in the spatial distribution of PAD and CLTI according to multiple deprivation. Our findings are in agreement with other studies which found a higher incidence of PAD in areas of greater socioeconomic disadvantage.^15,18,20^ However, our novel approach to geospatial mapping also allows for a visually simplified depiction of prevalence variation for PAD and CLTI for small communities with similar deprivation exposure. Further, we found hot and cold spot clusters occur across all deprivation quintiles, suggesting, in this population, those in less deprived areas are not fully afforded protection from developing PAD or CLTI.

We employed the SIMD, which captures a wide set of deprivation determinants ranging from housing, crime, education, and health (which incorporates comorbidities, alcohol, and drug misuse). Multiple deprivation may act to drive health disparities among communities through multiple mechanisms.^21^ Complex and long causal pathways and biological mechanisms may involve health behaviours such as smoking, obesity, and physical activity as key mediators for PAD and CLTI in people with diabetes. Importantly, the creation and persistence of health disparities are associated with social and environmental health determinants and are a cumulative risk for cardiovascular disease.^22^ For example, high levels of smoking in Greater Glasgow are attributable to lower socioeconomic status and smoking exhibits a strong social patterning.^23–25^ In Scotland, those in the most deprived areas have the highest non-attendance in primary care and have the worst mortality outcomes.^26^ This may have a negative effect on engagement with cardiovascular preventative interventions (e.g. statin or exercise therapy). Foster and colleagues (2018) have identified emerging adverse health behaviours in a large Scottish population, including that of sleep duration and high television viewing time, in addition to more established risk factors such as smoking, poor dietary habits, excess alcohol consumption, and physical inactivity.^25^ These behaviours were found in the most deprived populations and associated with higher cardiovascular-related mortality.^25^ Our observed clustering may reflect pull-down effects whereby concentrations of the most deprived neighbourhoods serve to deepen poor health behaviours and culture, and provide few resources to be drawn on.

### Strengths and limitations

The retrospective design of this study cannot account for bidirectional causation in explaining these findings. However, our findings reinforce previous studies that have demonstrated the association of PAD and CLTI in patients at socioeconomic disadvantage.^15,18,20^ In addition, the hot spots formed are consistent with Glasgow’s spatial deprivation profile.^27^ Coding errors present a constant challenge in capturing accurate clinical data. Hussey and colleagues (2016) carried out an evaluation of the quality of abdominal aortic aneurysm data and mortality capture in SMR-01 in Greater Glasgow and Clyde. They uncovered multiple coding errors with systematic bias.^28^ Inaccuracies in administrative datasets are unavoidable. However, a key strength to this study is the capture of a large study population using data linkage over a 13.6-year period, which offers some degree of rigour. We highlight that some lifestyle factors, such as smoking status, are not robustly captured in SCI-Diabetes, which is demonstrated by the high level of missing data reported for this variable. Importantly, SCI-Diabetes is a validated national clinical registry which gives almost complete population coverage for all individuals diagnosed with diabetes (99% coverage) across the health board.^29^ Additionally, we exploited small area geographical techniques to test the association with SIMD. Heterogeneity was ensured as each data zone boundary hosts ~760 inhabitants with similar exposures to multiple deprivation. This delivers a more granular overview of geographical variability. One drawback to this technique is that although data zones are built to represent small populations and physical boundaries with a fairly steady population size over the study period, we are unable to account for potential migration or relocation changes. Another limitation is that the spatial model was unadjusted for other confounding factors, which may explain the association of SIMD and PAD and CLTI. The focus of the study was not on risk modelling or prognostics, but the use of spatial statistics on crude prevalence of PAD and CLTI and the association with social deprivation. We recognise that one such important confounder was ethnicity. In the UK, it has been established that important ethic minority groups tend to live in localities with greater levels of socioeconomic disadvantage. This may have contributed to the spatial patterning observed. We recognise this is a limitation of the study, as the spatial model was not adjusted to consider this. Despite this, this population has a particularly niche demographic pattern not reflected elsewhere. In Glasgow, proportionately higher levels of key ethnic minority groups including Pakistani, Chinese and Indian populaces have less exposure to multiple deprivation.^30^ Finally, Glasgow provides an ideal testbed to investigate this spatial model as it is well established that there are major inequalities for people developing diabetes in Scotland.^31^ Further to this, the west of Scotland’s central belt has particularly poor cardiovascular outcomes and greater levels of premature mortality. However, this is intensified in Glasgow city and its surrounding areas. This health board region has historically suffered significant deindustrialisation around the shipbuilding industry. Whilst we cannot account for the environmental contaminants associated with Glasgow’s history in heavy engineering, or other more recent pollutants, in explaining these poor health outcomes in this population, we can understand this has resulted in higher levels of concentrated deprivation than seen in the rest of Scotland.^27^

## Conclusion

In conclusion, we utilised a granular geospatial approach to capture the cluster patterning of PAD diagnoses and its association with social deprivation. Individuals with diabetes in the most deprived quintile were 5.6–7.9 times more likely to have PAD or CLTI diagnoses compared to the least deprived quintile. The inequity highlighted offers a unique public health opportunity to target both resources and education to reduce major adverse cardiovascular events in an ‘at-risk’ population.

## Clinical implications and future research

This study will help inform updated local clinical pathways with consideration of exposure to social deprivation in patients with diabetes who have a diagnosis of PAD and/or CLTI. In addition, this observational study may navigate where more empirical research is required in this field. Furthermore, it may aid future planning of diabetic lower limb services and resource utilisation.

## Supplemental Material

sj-docx-1-vmj-10.1177_1358863X20981132 – Supplemental material for Association of peripheral artery disease and chronic limb-threatening ischemia with socioeconomic deprivation in people with diabetes: A population data-linkage and geospatial analysis Click here for additional data file. Supplemental material, sj-docx-1-vmj-10.1177_1358863X20981132 for Association of peripheral artery disease and chronic limb-threatening ischemia with socioeconomic deprivation in people with diabetes: A population data-linkage and geospatial analysis by Joanne E Hurst, Peta Ellen Tehan, Keith Hussey and James Woodburn in Vascular Medicine
