# Supplementary material for: Association of peripheral artery disease and chronic limb-threatening ischemia with socioeconomic deprivation in people with diabetes: A population data-linkage and geospatial analysis
Source: Vasc Med. 2021 Jan 25;26(2):147–54. doi: 10.1177/1358863X20981132 (PMC8033436; doi:10.1177/1358863X20981132)
Supplement: sj-docx-1-vmj-10.1177_1358863X20981132 – Supplemental material for Association of peripheral artery disease and chronic limb-threatening ischemia with socioeconomic deprivation in people with diabetes: A population data-linkage and geospatial analysis [file sj-docx-1-vmj-10.1177_1358863X20981132.docx]

**ESM Fig. 1.**

**
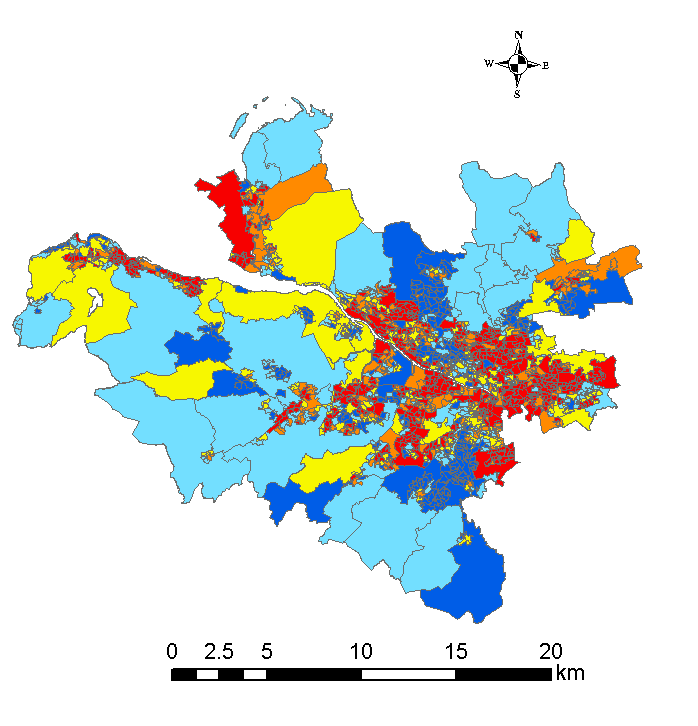
**

**
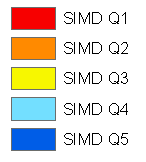
**

Data source: The 2016 SIMD map shape file is available on the Scottish Government website: <https://www2.gov.scot/Topics/Statistics/SIMD>

**ESM Fig 1.** Choropleth map indicating Scottish Index of Multiple Deprivation Quintile distribution across NHS Greater Glasgow and Clyde health board.
